# Clinical differences between small and large pheochromocytomas and paragangliomas

**DOI:** 10.3389/fendo.2023.1087506

**Published:** 2023-03-09

**Authors:** Lin Zhao, ZhiMao Li, Xu Meng, Hua Fan, ZengLei Zhang, ZhaoCai Zhang, YeCheng Liu, XianLiang Zhou, HuaDong Zhu

**Affiliations:** ^1^ Department of Cardiology, Fuwai Hospital, Chinese Academy of Medical Sciences and Peking Union Medical College/National Center for Cardiovascular Disease, Beijing, China; ^2^ Emergency Department, State Key Laboratory of Complex Severe and Rare Diseases, Peking Union Medical College Hospital, Chinese Academy of Medical Science and Peking Union Medical College, Beijing, China; ^3^ Department of Urology, State Key Laboratory of Complex Severe and Rare Diseases, Peking Union Medical College Hospital, Chinese Academy of Medical Science and Peking Union Medical College, Beijing, China; ^4^ Department of Critical Care Medicine, The Second Affiliated Hospital, Zhejiang University School of Medicine, Hangzhou, Zhejiang, China

**Keywords:** pheochromocytoma, paraganglioma, catecholamine, clinical features, tumor diameter

## Abstract

**Background:**

Pheochromocytomas and paragangliomas (PPGLs) are neuroendocrine tumors, most of which are characterized by the release of catecholamine, and range in diameters from less than 1 cm to 10 cm or more. However, knowledge of the differences in clinical features between small and large PPGLs is insufficient.

**Methods:**

A retrospective analysis of patients with PPGLs treated at our institution between January 2018 and June 2020 was performed. The clinical characteristics of patients were investigated, and comparisons were made between patients with large and small PPGLs. The logistic regression analysis was used to confirm the risk factors, and the receiver operating characteristic curve was used to evaluate the diagnostic performance of the variables.

**Results:**

Totally 263 patients were included, including 110 patients in small tumor group and 153 patients in large tumor group. There were more male patients in the large tumor group (p=0.009). More patients had hypertension (p<0.001) and diabetes (p=0.002) in the large tumor group. The 24-h urinary epinephrine (24hU-E) (p < 0.001) and 24-h urinary norepinephrine (24hU-NE) (p=0.002) concentrations were higher in the large tumor group. In terms of tumor location, adrenal-PPGLs were more frequent in the large tumor group (p<0.001). Multivariate logistic regression analysis showed that male sex [odds ratio (OR): 2.871, 95% confidence interval (CI): 1.444–5.711, p=0.003], 24hU-E concentrations (OR: 1.025, 95% CI:1.004–1.047, p=0.020), 24hU-NE concentrations (OR: 1.002, 95%CI: 1.001–1.004, p=0.045), and adrenal-PPGLs (OR: 2.510, 95% CI:1.256–5.018, p=0.009) were positive risk factors for large tumors. Taking above variables into the same model, the area under the receiver operating characteristic curve of the model for predicting the large tumor was 0.772 (95% CI: 0.706–0.834). After the short-term follow-up, there was no significant difference in tumor recurrence between the two groups (p=0.681).

**Conclusions:**

Significant differences in numerous clinical characteristics exist between large and small PPGLs. The male patients were more likely to be with large tumors, and such tumors were more likely to reside on the adrenal glands. Catecholamine measurements also help predict tumor size of PPGLs. Clinical decision-making will benefit from this information.

## 1 Introduction

Pheochromocytomas and paragangliomas (PPGLs) are neuroendocrine tumors, most of which are characterized by the production of catecholamines. Adrenal PPGLs originate from adrenomedullary chromaffin cells, and extra-adrenal PPGLs arise from extra-adrenal chromaffin cells of the sympathetic paravertebral ganglia located in the thorax, abdomen, pelvis, as well as from parasympathetic ganglia (1). The combined incidence of PPGLs is approximately 0.57 cases per 100,000 person years (2). These tumors commonly cause hyperadrenergic symptoms such as hypertension, headaches, palpitations, and sweating (3). There is a wide range of sizes for PPGLs, ranging from less than 1 to 10 cm or more in diameter (4). Investigations of the imaging differences between large and small PPGLs have been conducted, for example, Reinig et al. (5) and Kim et al. (4) found that smaller PPGLs tended to be homogeneous, whereas larger tumors were heterogeneous because of hemorrhage and necrosis. However, insufficient data are available to determine whether small and large PPGLs differ in clinical features. Therefore, this study was aim to evaluate such differences.

## 2 Materials and methods

### 2.1 Study population

**Figure 1:**
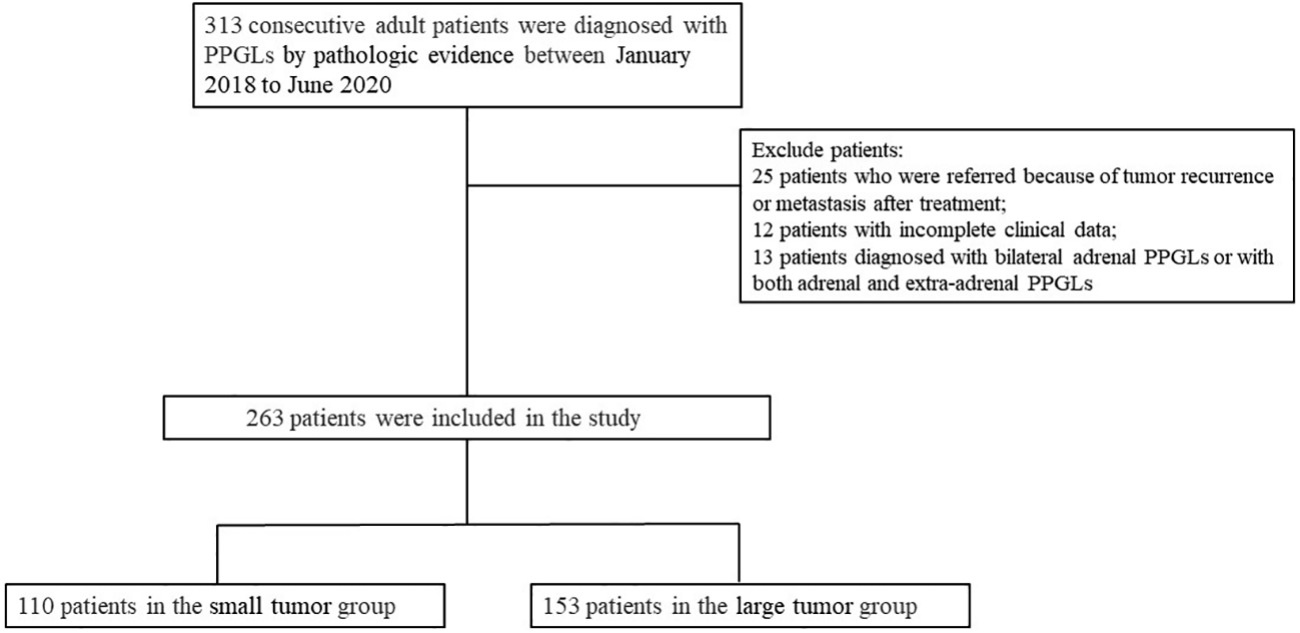
Flow chart showing the selection of patients. PPGLs: pheochromocytomas and paragangliomas.

All consecutive adult patients (n = 313) with PPGLs who were treated at Peking Union Medical College Hospital, Beijing, China, between January 2018 and June 2020 were enrolled. All patients with PPGLs included in our study were surgically treated and diagnosed by surgical pathology. We excluded 25 patients who were referred because of tumor recurrence or metastasis after treatment and excluded 12 with incomplete clinical information. The study also excluded patients diagnosed with bilateral adrenal PPGLs (n = 6) or with concurrent adrenal PPGLs and extra-adrenal PPGLs (n = 7) upon their first visit, because it was not possible to identify which tumors were functional. Therefore, 263 patients were included in the analysis. **Figure 1** shows a flowchart describing patient selection. The Ethics Committee of Peking Union Medical College Hospital approved the study, which was also conducted in accordance with the provisions of the Declaration of Helsinki. The requirement for informed consent was waived because of the retrospective nature of the study, and all data were anonymized and deidentified.

### 2.2 Clinical assessment

We collected and analyzed retrospective data on patients’ clinical histories, biochemical test results, and surgical pathological findings. Hypertension was defined (6) as follows: 1) systolic blood pressure (SBP) in the office ≥140 mmHg and/or diastolic BP (DBP) ≥90 mmHg following repeated examinations; or 2) ambulatory BP monitoring results showing the averages of SBP/DBP of 24 h ≥130 and or ≥80 mmHg; or 3) home SBP ≥135 mmHg and/or home DBP ≥85 mmHg; or 4) an existing diagnosis of hypertension with an established antihypertension diet or treatment. A variety of hypertension patterns were present in PPGLs, including sustained, paroxysmal, and mixed patterns (3). Hypertension was diagnosed according to preoperative BP data. The definition of diabetes was as follows: 1) in repeated tests of asymptomatic patients, during a 75-h oral glucose tolerance test, a fasting plasma glucose was ≥7.0 mmol/L, or a 2-h plasma glucose value ≥11.1 mmol/L; or HbA1c ≥6.5%; or 2) in patients with classic hyperglycemia symptoms, the random plasma glucose level was ≥11.1 mmol/L; or 3) an existing diagnosis of diabetes with an established hypoglycemic diet or treatment (7). We allocated patients into two groups according to a cutoff value determined according to the typical sizes of malignant adrenal tumors. A lesion over 4 cm in diameter has a 70% chance of becoming malignant (8), and we therefore chose a diameter of 4cm as the cutoff value. Tumor diameters were determined according to the histopathological findings using tissue specimens. Tumors with diameter > 4cm were considered as large tumors and tumors with diameter≤ 4cm were considered as small tumors. Measurements of 24-h urinary catecholamines were conducted using the high-performance liquid chromatography-mass spectrometry. Data for all laboratory indicators were acquired upon the patient’s first visit to our medical institution. The minimum follow-up time was 6 months after surgery. Follow-up results of all patients were obtained through outpatient records and telephone calls.

### 2.3 Statistical analysis

Continuous values were reported as the mean ± standard or median (25th, 75th percentiles). Student’s t-tests or rank-sum tests were used to compare continuous variables between groups. Categorical variables were represented as numbers (percentages), and Pearson’s chi-square or Fisher’s exact tests were used to evaluate the significance of differences. Multivariate logistic regression included parameters with p < 0.1 in univariate logistic regressions. SPSS statistical software, version 25.0 (IBM Corp. Armonk, USA) was used to perform all analyses. GraphPad Prism 8.0 (GraphPad Software Corp. CA, USA) was used to analyze receiver operating characteristic (ROC) curves. Statistical significance was defined as two-sided p values < 0.05.

## 3 Results

### 3.1 Characteristics of the whole cohort

The clinical characteristics of patients with PPGLs are shown in **Table 1**. We included 263 patients in the analysis, including 119 patients with adrenal PPGLs (45.2%) and 144 patients with extra-adrenal PPGLs (54.8%). Among patients with extra-adrenal PPGLs, 72 patients had tumors located in the head and neck, and 72 patients had tumors located in the trunk. The mean age of the subjects was 45.9 ± 12.9 years. Men accounted for 46.8% of the cohort. Sixty-one (23.2%) patients had diabetes, and 142 (54.0%) had hypertension. Eighty-eight patients (33.5%) had sustained hypertension, 48 (18.3%) had paroxysmal hypertension, 6 (2.3%) had mixed hypertension. Thirty-five percent of patients reported dizziness or headache, 33.1% reported palpitations, 25.1% reported excessive sweating, 7.2% reported nausea or vomiting, and 6.5% reported PPGL crisis.

**Table 1:** Clinical characteristics of patients with PPGLs at initial presentation.

variable	All patients (n=263)
Age, years(n=263)	45.9 ± 12.9
Male, % (n=263)	123(46.8)
BMI, kg/m^2^(n=263)	24.2 ± 3.3
Diabetes, % (n=263)	61(23.2)
Hypertension, %(n=263)	142(54.0)
Patterns of hypertension	
Sustained, %	88(33.5)
Paroxysmal, %	48(18.3)
Mixed, %	6(2.3)
Symptoms(n=263)	
Dizziness or headache, %	92(35.0)
Palpitations, %	87(33.1)
Excessive sweating, %	66(25.1)
Nausea or vomiting, %	19(7.2)
PPGL crisis, %	17(6.5)
24hU-E, μg/24h(n=205)	4.2(2.8, 16.6)
24hU-NE, μg/24h (n=205)	51.2(32.1, 182.9)
24hU-DA, μg/24h (n=205)	232.3(186.3,297.8)
Adrenal PPGLs, % (n=263)	119(45.2)
Extra-adrenal PPGLs, % (n=263)	144(54.8)
Tumor diameters (cm) (n=263)	5.0(3.2, 6.0)

BMI, body mass index; PPGLs, pheochromocytomas and paragangliomas; 24hU-E, 24-h urine epinephrine; 24hU-NE, 24-h urine norepinephrine; 24hU-DA, 24-hour urine dopamine.

Reference ranges: 24-hour urinary epinephrine: 1.74–6.42 μg/24 h; 24-hour urinary norepinephrine: 16.69–40.65 μg/24 h; 24-hour urinary dopamine: 120.93–330.59 μg/24 h.

### 3.2 Characteristics of patients between the two groups

During the whole study, 110 patients were in the small tumor group, and 153 patients were in the large tumor group. The clinical characteristics of patients in the two groups are shown in **Table 2**. Age and body mass index were not significantly different between the groups. Compared with small tumor group, the proportion of men in the large tumor group was higher (53.6% vs 37.3%, p = 0.009), and more patients in the large tumor group had hypertension and diabetes (64.1% vs 40.0%, p < 0.001; and 30.1% vs 13.6%, p = 0.002, respectively). Palpitations, excessive sweating, and nausea or vomiting were more likely to be experienced in the large tumor group, while the frequencies of dizziness or headache and PPGL crisis were not significantly different between the two groups. The levels of 24-h urinary epinephrine (24hU-E) (p*<* 0.001) and 24-h urinary norepinephrine (24hU-NE) (p = 0.002) in the large tumor group were also higher. There was no significant difference in 24-h urinary dopamine levels between groups (p = 0.063). Adrenal PPGLs were more frequent to be found in the large tumor group (61.4% vs 22.7%, p < 0.001), and extra-adrenal PPGLs were more frequent to be found in the small tumor group (77.3% vs 38.6%, p < 0.001).

**Table 2:** Clinical characteristics of patients between the two groups.

variable	Small tumor group (n=110)	Large tumor group (n=153)	P value
Age, years(n=263)	45.6 ± 13.3	46.1 ± 12.6	0.766
Male, % (n=263)	41(37.3)	82(53.6)	0.009
BMI, kg/m^2^(n=263)	24.0 ± 3.5	24.3 ± 3.2	0.505
Diabetes, % (n=263)	15(13.6)	46(30.1)	0.002
Hypertension, %(n=263)	44(40.0)	98(64.1)	<0.001
Patterns of hypertension			
Sustained, %	24(21.8)	64(41.8)	0.001
Paroxysmal, %	19(17.3)	29(19.0)	0.728
Mixed, %	1(0.9)	5(3.3)	0.398
Symptoms(n=263)			
Dizziness or headache, %	33(30.0)	59(38.6)	0.151
Palpitations, %	24(21.8)	63(41.2)	0.001
Excessive sweating, %	16(14.5)	50(32.7)	0.001
Nausea or vomiting, %	3(2.7)	16(10.5)	0.017
PPGL crisis, %	5(4.5)	12(7.8)	0.283
24hU-E, μg/24h(n=205)	3.6(2.5,5.0)	4.8(3.0,29.0)	<0.001
24hU-NE, μg/24h (n=205)	36.1(25.8,149.9)	70.5(36.2,197.3)	0.002
24hU-DA, μg/24h (n=205)	218.8(180.2,257.2)	237.0(188.3,306.1)	0.063
Adrenal PPGLs, % (n=263)	25(22.7)	94(61.4)	<0.001
Extra-adrenal PPGLs, % (n=263)	85(77.3)	59(38.6)	<0.001

BMI, body mass index; PPGLs, pheochromocytomas and paragangliomas; 24hU-E, 24-h urine epinephrine; 24hU-NE, 24-h urine norepinephrine; 24hU-DA, 24-hour urine dopamine.

Reference ranges: 24-hour urinary epinephrine: 1.74–6.42 μg/24 h; 24-hour urinary norepinephrine: 16.69–40.65 μg/24 h; 24-hour urinary dopamine: 120.93–330.59 μg/24 h.

Multivariate logistic regression analysis was used to identify risk factors of large tumors in patients with PPGLs. The clinical symptoms in patients with PPGLs were related to the secretion of catecholamines, therefore, interactions among these parameters were possible. Consequently, only catecholamine concentrations were included in the logistic regression analysis. According to the results of the univariate logistic regression analysis (**Table 3**), sex, diabetes, hypertension, 24hU-E concentrations, 24hU-NE concentrations, 24hU-dopamine concentrations and tumor locations were included in the multivariate logistic regression analysis. The result showed that male sex [odds ratio (OR): 2.871, 95% confidence interval (CI): 1.444–5.711, p = 0.003], 24hU-E concentrations (OR: 1.025, 95% CI: 1.004–1.047, p = 0.020), 24hU-NE concentrations (OR: 1.002, 95% CI: 1.001–1.004, p = 0.045), and adrenal PPGLs (OR: 2.510, 95% CI: 1.256–5.018, p = 0.009) were positive risk factors for large tumors in patients with PPGLs.

**Table 3:** Results of the logistic regression analysis.

variable	Univariate logistic regression analysis			Multivariate logistic regression analysis		
	p	OR	95%CI	p	OR	95%CI
Age*	0.765	1.003	0.984-1.022			
Male**	0.009	1.944	1.179-3.206	0.003	2.871	1.444-5.711
Diabetes**	0.002	2.723	1.429-5.189	0.528	0.741	0.291-1.883
Hypertension**	<0.001	2.673	1.614-4.427	0.994	0.997	0.479-2.078
BMI*	0.504	1.026	0.962-1.105			
24hU-E*	0.005	1.033	1.01-1.057	0.020	1.025	1.004-1.047
24hU-NE*	0.017	1.002	1.001-1.004	0.045	1.002	1.001-1.004
24hU-DA*	0.044	1.003	1-1.006	0.147	1.001	0.999-1.003
Adrenal PPGLs **	<0.001	5.417	3.119-9.409	0.009	2.510	1.256-5.018

BMI, body mass index; PPGLs, pheochromocytomas and paragangliomas; 24hU-E, 24-h urine epinephrine; 24hU-NE, 24-h urine norepinephrine; 24hU-DA, 24-hour urine dopamine

* For quantitative variables, OR is presented for per unit increase of the given variable.

** For qualitative variable, OR is presented for presence of that variable.

**Figure 2:**
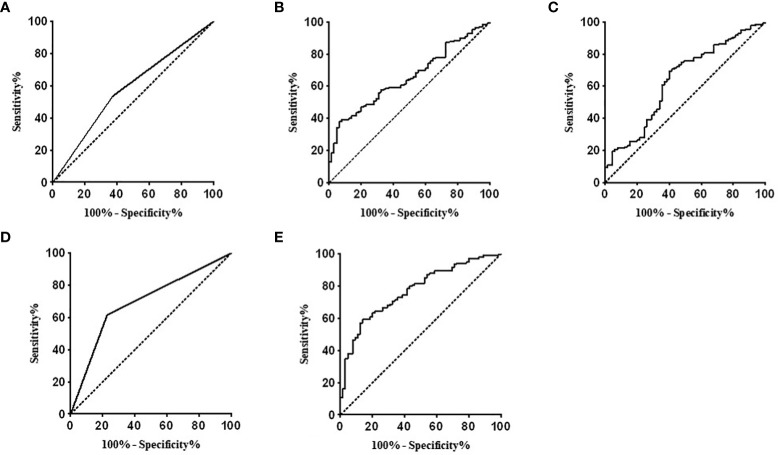
Receiver operating characteristic curve analysis evaluating the diagnostic performance for tumor diameter in patients with PPGLs. **(A)**The AUC of the male for predicting the large tumor was 0.582 (95% CI: 0.512–0.651, p=0.024). **(B)** The AUC of the 24U-E concentrations for predicting the large tumor was 0.656 (95% CI: 0.581–0.731, p<0.001). **(C)** The AUC of the 24hU-NE concentrations for predicting the large tumor was 0.637 (95% CI: 0.554 – 0.720, p = 0.002). **(D)** The AUC of the adrenal PPGLs for predicting the large tumor was 0.694 (95% CI: 0.629–0.758, p<0.001). **(E)** The AUC of the whole model for predicting the large tumor was 0.772 (95% CI: 0.706–0.834, p < 0.001). PPGLs, pheochromocytomas and paragangliomas; AUC, area under the curve; 24hU-E, 24-hour urinary epinephrine; 24hU-NE, 24-hour urinary norepinephrine.

The ROC curve analysis was used to evaluate the diagnostic performance of the variables. The area under the ROC curve (AUC) of males for predicting the large tumor was 0.582 (95% CI: 0.512–0.651, p = 0.024); the AUC of the 24hU-E concentrations for predicting the large tumor was 0.656 (95% CI: 0.581–0.731, p < 0.001); the AUC of the 24hU-NE concentrations was 0.637 (95% CI: 0.554–0.720, p = 0.002); the AUC of adrenal PPGLs was 0.694 (95% CI: 0.629–0.758, p < 0.001). Taking gender, 24hU-E concentrations 24hU-NE concentrations, and adrenal PPGLs into account in the same model, the AUC of the model for predicting the large tumor was 0.772 (95% CI: 0.706–0.834, p < 0.001) (**Figure 2**). A total of 212 patients in our study underwent regular imaging review after surgery, of which 90 were with the preoperative small tumor group and 122 were with the preoperative large tumor group. After the mean follow-up of 20.2 ± 11.7 months, there was no significant difference in recurrence between the two groups (4.4% vs. 2.5%, p = 0.681).

## 4 Discussion

In this study, we found that the tumor diameters in male patients with PPGLs were more likely to exceed 4 cm, and high concentrations of catecholamines could predict large PPGLs. Furthermore, large tumors were more likely to reside on the adrenal glands. After the short-term follow-up, there was no significant difference in tumor recurrence between the two groups. Our findings provide an important basis for further understanding of the clinical characteristics of PPGLs, the risk stratification of patients with PPGLs, and developing a reasonable clinical screening and follow-up plan.

Published studies have described the relationship between catecholamines and tumor diameter in patients with PPGLs. In Falhammar et al.’s study (9), urine norepinephrine/plasma normetanephrine levels and tumor size were positively correlated. Additionally, Guerrero et al. (10) found a direct, significant correlation between tumor size and catecholamine hormone levels independent of clinical presentation; and when excluding confounding factors, there was a stronger linear correlation between them. Furthermore, hormone levels vary greatly among all tumor sizes, with smaller tumors exhibiting a lower tendency to secrete high levels of catecholamines. The results of Eisenhofer’s study (11) also indicated that tumor diameter correlated positively with summed 24h urinary normetanephrine and metanephrine (p<0.001). In addition to the strong correlation between tumor diameter and plasma or urinary deconjugated metanephrines, there was also a significant positive relationship between tumor diameter and urinary or plasma catecholamines (p<0.001). Since most patients did not have the results of plasma free metanephrines or urinary fractionated metanephrines in this study, which provide higher sensitivity and specificity (1), we couldn’t get the relationship between them and tumor diameter. The relationship between the 24hU-catecholamines and tumor diameter in the present study is in accordance with the findings of the studies above. This data in the present study indicates that urinary catecholamine concentrations can serve as a predictor of tumor size. Accordingly, we are able to predict the tumor diameters prospectively. A prediction like this, may be helpful during subsequent imaging procedures to confirm the localization of the tumor. It is increasingly necessary for laboratory medicine to be integrated into making diagnose, and particularly important to provide guidance regarding testing procedures, interpretations, and follow-ups. One example where such guidance may be especially useful is the laboratory diagnosis of PPGLs.

As for the tumor diameters of adrenal and extra-adrenal PPGLs, in this study, adrenal PPGLs were more prone to be large tumors, which is consistent with the findings of others (12, 13). Goffredo et al. compared clinical characteristics between malignant adrenal PPGLs and extra-adrenal PPGLs, they found adrenal PPGLs were larger than extra-adrenal PPGLs (mean size 7.7 vs. 4.5 cm, p = 0.001), and larger tumor size was also associated with greater mortality (12). Similarly, in a study of describing baseline characteristics of patients with malignant PPGLs, Hamidi et al. (13) found that compared with extra-adrenal PPGLs, adrenal PPGLs were larger (median size 9.0 cm vs 5.8 cm, p < 0.0001) and were more frequently functional (91% vs 72%, p = 0.0001); they also reported that older age at primary diagnosis, larger tumor size, and synchronous metastases were independent factors for the shorter survival. Other studies have also reported that larger or heavier tumors are strongly associated with malignant disease and mortality (14, 15). In the present study, there was no significant difference in tumor recurrence between the groups after the short-term follow-up. The possible reasons for this maybe that we excluded patients diagnosed with metastatic PPGLs at first visit in our hospital before analysis, and as metastatic PPGLs often become evident several years after initial diagnosis, this may be also due in part to the short-term follow-up.

In this study, men sex was more likely to harbor large tumors, in fact, previous studies have also found that men sex was associated with the possibility of malignancy (13, 16). In a large retrospective cohort of patients with adrenal tumors, Iñiguez-Ariza et al. (16) found that one of the factors that predicted a malignant adrenal mass was male sex. Hamidi et al. (13) reported that there was a significant association between male sex and shorter survival (p = 0.014) in patients with malignant PPGLs. This suggests that compared with female patients, male patients may require more attention and close clinical follow-up to reduce the occurrence of adverse outcomes.

There are several limitations in this study. Firstly, this was a single-center retrospective study, and the results should be generalized with caution. Secondly, not all patients’ urinary catecholamines were measured. Thirdly, due to the lack of genetic screening results, we couldn’t get relationship between genotype and tumor diameter. To evaluate the exact clinical features of small and large PPGLs, a prospective cohort study is required.

## 5 Conclusion

Significant differences in numerous clinical characteristics exist between large and small PPGLs. Male patients with PPGLs were more likely to be with large tumors, and large tumors were more likely to reside on the adrenal glands. Furthermore, catecholamine measurements not only provide information for predicting the presence or absence of PPGLs, but also help predict tumor size if PPGLs are present. It may be useful to make clinical decisions according to this information, and clinicians must be aware of the clinical features of PPGLs since early identification of a can be life-saving.

## Data availability statement

The original contributions presented in the study are included in the article/supplementary material. Further inquiries can be directed to the corresponding authors.

## Ethics statement

The studies involving human participants were reviewed and approved by the Ethics Committee of Peking Union Medical College Hospital. Written informed consent for participation was not required for this study in accordance with the national legislation and the institutional requirements.

## Author contributions

LZ, YCL and XLZ conceptualized and designed the study. LZ, ZML and XM provided analyzed and interpreted the data. LZ, ZML, HF, ZLZ and ZCZ provided statistical support, including data collection and assembly. YCL, XLZ and HDZ reviewed the framework and content of the discussion. All authors contributed to the article and approved the submitted version.
